# A near-complete genome assembly of *Monochamus alternatus* a major vector beetle of pinewood nematode

**DOI:** 10.1038/s41597-024-03150-1

**Published:** 2024-03-26

**Authors:** Longsheng Xing, Bo Liu, Dunyang Yu, Xuan Tang, Jianghua Sun, Bin Zhang

**Affiliations:** 1 College of Life Science/Hebei Basic Science Center for Biotic Interactions, Institute of Life Science and Green Development, Hebei University, Baoding, 071002 China; 2 Shenzhen Branch, Guangdong Laboratory for Lingnan Modern Agriculture, Genome Analysis Laboratory of the Ministry of Agriculture and Rural Affairs, Agricultural Genomics Institute at Shenzhen, Chinese Academy of Agricultural Sciences, Shenzhen, 518120 China

**Keywords:** Evolutionary ecology, Molecular ecology

## Abstract

The Japanese sawyer beetle, *Monochamus alternatus*, is not only one of the most important wood boring pest itself, but also a major vector of the invasive pinewood nematode (PWN), which is the causal agent of the devastative pine wilt disease (PWD) and threats the global pine forest. Here, we present a near-complete genome of *M. alternatus* at the chromosome level. The assembled genome was 792.05 Mb with contig N50 length of 55.99 Mb, which is the largest N50 size among the sequenced Coleoptera insects currently. 99.57% of sequence was anchored onto ten pseudochromosomes (one X-chromosome and nine autosomes), and the final genome harbored only 13 gaps. BUSCO evaluation revealed the presence of 99.0% of complete core genes. Thus, our genome assembly represented the highest-contiguity genome assembly as well as high completeness in insects so far. We identified 20,471 protein-coding genes, of which 20,070 (98.04%) were functionally annotated. The genome assembly of *M. alternatus* provides a valuable resource for exploring the evolution of the symbiosis between PWN and the vector insects.

## Background & Summary

Vector-borne plant diseases widely occur and cause severe ecological and economic losses in agricultural and forestry ecosystem. Vector insects play particularly important roles in the evolution of the pathogen dispersion and pathogenesis^1^. The plant parasitic nematode *Bursaphelenchus xylophilus*, also known as pine wood nematode (PWN), is the causal agent of the devastative pine wilt disease (PWD) and threats the global pine forest^2,3^. The transmission of PWN from dead pine trees to susceptible, live pine trees exclusively relied on the vector beetles belonging to species of the genus *Monochamus* (Coleoptera: Cerambycidae)^4^. During the invasion history of PWN, its vector species also shifted with the geographic locations. The primary vector in North America, native region of PWN, is *Monochamus carolinensis*, then changed to *M. alternatus* and *M. salturatis* in Asia and *M. galloprovincialis* in Europe, the invasive regions of PWN^5^. The vector species shift thus greatly contributes to the evolutionary ecology of PWD^6^. However, the underlying molecular mechanism is still far unknown due to few genomic resources of these vector insects^7,8^.

The Japanese swayer beetle, *M. alternatus*, is not only a main vector of the invasive PWN, but also one of the most important wood boring pest itself across East Asia such as China, South Korea and Japan, where is the place with the most serious PWD epidemic damage^9–11^. This vector beetle and PWN has formed a close symbiosis based on their high synchronization of life cycle, mediated by the chemical signals^11–13^. Specifically, the *M. alternatus* beetles prefer to select the weakened or dying trees with PWN infection to oviposit and complete the development of their offspring. The third-stage juveniles (L_III_) of PWN are attracted by specific terpenes produced by mature insect larvae and aggregate around pupal chambers in diseased trees^12^, and fourth-stage juveniles (L_IV_) are attracted into the trachea of newly emerged adults by ascarosides secreted by the beetles^13^. The newly eclosed beetle should have a maturation feeding in healthy trees. The nematode then departs from the spiracles driven by CO_2_ enhanced by feeding behavior and invades new healthy trees via the feeding wounds^14,15^, thus starting a new cycle of infection, propagation and dispersal. While the chemical signals among the symbiosis have been well characterized, the molecular mechanism of the chemical communications remains elusive. Furthermore, as with most vector-borne diseases, vector control is the key to manage those diseases efficiently. Unfortunately, there is still lack of effective and efficient control method against this vector beetle, therefore, a high-quality reference genome is needed for both further understanding this symbiosis and its maintenance as well as new control approaches, such as genetic-engineered management strategy.

Recently, Gao *et al*. reported a chromosome-level genome assembly of *M. alternatus* based on Nanopore sequencing technology^8^. Here, we constructed a high-quality chromosome-scale genome of *M. alternatus* through combining Pacific Biosciences (PacBio) high-fidelity (HiFi), high-throughput chromosome conformation capture (Hi-C), and Illumina short-read sequencing data. Subsequently, we performed structural and functional annotation of the assembled genome through integrating transcriptome data from different tissues of *M. alternatus*. The high-quality reference genome of *M. alternatus* provides a valuable resource for exploring the evolution of coleopteran insects and the interaction mechanism between PWN and its vector insects.

**Fig. 1:**
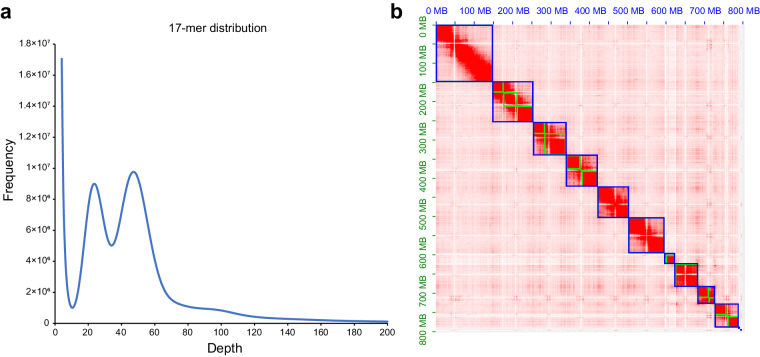
Genome size estimation and heatmap of genome-wide Hi-C interaction. **(a)** K-mer frequency analysis was performed for genome size estimation of *M. alternatus* using GCE (v1.0.2) based on Illumina genome sequencing data. The second peak with depth at 48 represents the main peak, and the first peak with depth at 24 indicates the heterozygous peak. **(b)** The heatmap shows the scaffolding result of the *M. alternatus* genome based on the juicer and 3ddna pipeline. The first ten blue rectangles represents ten pseudochromosomes.

A total of 99.27 Gb (127.81×) of HiFi reads (Table 1) were used to generate the primary genome assembly. Furthermore, 145.39 Gb (187.19×) of Hi-C data (Table 1) was used to anchor contigs to chromosome-level genome using the juicer and 3D-DNA pipeline. The final assembled genome is 792.5 Mb, which was very close to the estimated genome size (776.7 Mb) (Fig. 1a; Table 2) based on the distribution of k-mer frequencies, with a contig N50 size of 55.99 Mb and a scaffold N50 size of 86.21 Mb. Based on Hi-C data, 99.57% of genome sequence was successfully anchored onto ten pseudochromosomes (Fig. 1b). Realignment of Illumina genome sequencing and RNA-seq reads to the reference genome achieved average mapping rates of 98.32% and 95.53% (Table S1), respectively. MUMmer-based genome alignment indicated that our genome assembly exhibited 1:1 synteny relationship with the closely related species *M. saltuarius* (Fig. S1), and chromosome 7 of *M. alternatus* was determined as the X chromosome based on chromosomal synteny. BUSCO v5.2.2^16^ was used to evaluate the completeness of the genome assembly of *M. alternatus* based on the insect_odb10 dataset. The results showed that 99.0% of complete BUSCOs were successfully captured by our genome assembly, including 98.3% of single-copy and 0.7% of duplicated BUSCOs (Table 2).

**Table 1:** Summary of genome sequencing data for *Monochamus alternatus*.

Sequencing strategy	Platform	Usage	Insertion size	Total data (Gb)	Sequence coverage (×)
Short-reads	Illumina	Genome survey	250 bp	43.05	55.43
HiFi	PacBio Sequel II	Assembly	15 kb	99.27	127.81
Hi-C	Illumina	Hi-C assembly	350 bp	145.39	187.19

**Table 2:** Summary statistics of *Monochamus alternatus* genome assembly.

	*Monochamus alternatus*
**Genome assembly**	
Estimated genome size (Mb)	776.7
Assembled genome size (Mb)	792.05
Contig N50 (Mb)	55.99
Number of contigs	33
Scaffold N50 (Mb)	86.21
Number of scaffolds	14
GC content (%)	32.37
Anchoring rate (%)	98%
Complete BUSCO of genome assembly	99.00%
**Gene annotation**	
Number of protein-coding genes	20,471
Average gene length (bp)	8161.45
Average CDS length (bp)	1175.53
Average exon length (bp)	282.41
Average intron length (bp)	2207.48
Average exon number	4.16
Complete BUSCO of gene annotations	97.60%

**Fig. 2:**
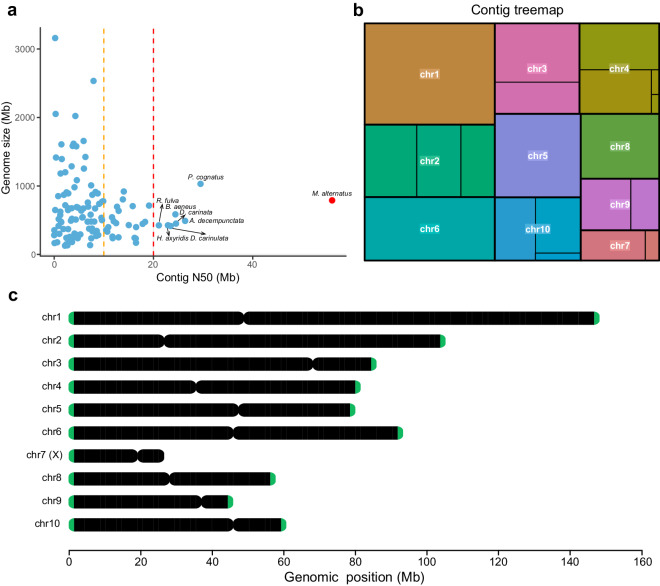
Comparison of the *M. alternatus* genome assembly with other sequenced Coleoptera insects and contig size tree map of *Monochamus alternatus* genome. (**a)** Scatter plot showing the contig N50 distribution of genome assemblies for *M. alternatus* and other Coleoptera insects publicly available. The assembly reports for Coleoptera insects were collected from NCBI datasets, and only chromosome-level genome assemblies were used for comparison. X-axis indicates the contig N50 size in megabases (Mb), and Y-axis denotes the genome size in Mb. The vertical dotted lines in orange and red represent the cutoff of 10 Mb and 20 Mb, respectively. Currently, a total of seven public Coleoptera insect genome assemblies showed contig N50 size ≥ 20 Mb. Compared with these available genome assemblies of Coleoptera insects, the *M. alternatus* genome assembly exhibited the highest contiguity level. **(b)** Tree map representation of the distribution of chromosome sizes and corresponding contig sizes. Four chromosomes chr1, chr5, chr6, and chr8 were composed of a single contig, the remaining six chromosomes harbored no more than three gaps for each. **(c)** Identification of telomeres and centromeres in the *M. alternatus* genome assembly. The telomeres were identified using the TeloExplorer module within quarTeT with ‘AACCT’ as the repeat monomer. The CentroMiner module within quarTeT was used for identification of centromeric regions.

Additionally, we made a comparison of contig size between *M. alternatus* and other coleopteran insects with publicly available genome assemblies in NCBI database. Compared with 116 other chromosome-level genome assemblies of Coleoptera insects, the genome assembly of *M. alternatus* showed the highest quality among the coleopteran insects at least in terms of contig N50 size (Fig. 2a; Table S2). Tree map representation was utilized to display contig size and gap number for each chromosome (Fig. 2b), indicating that four chromosomes (i.e. chr1, chr5, chr6, and chr8) were gap-free and the remaining six chromosomes harbored no more than three gaps for each. Moreover, we examined whether telomeres and centromeres were present in our assembled genome. The results showed that telomeric regions could be detected on both ends of nine chromosomes, and the telomeric region was identified on the single end of chromosome 7 (Fig. 2c; Table S3). As with centromeres, one candidate centromeric region was identified for each of ten chromosomes (Fig. 2c; Table S4). Together, we obtained a high-quality genome assembly of *M. alternatus* with high contiguity and high completeness.

Besides, we made a comparison of quality metrics between two genome assemblies generated by PacBio HiFi (hereafter referred to as HiFi assembly) and Nanopore sequencing technologies (hereafter referred to as Nanopore assembly). Firstly, we recalculated the N50 metrics of scaffold-level and contig-level genome using the same software assembly-stats to exclude the bias caused by different approaches. The HiFi assembly possessed higher quality in contig N50 size (55.99 Mb) compared with the Nanopore assembly (15.77 Mb) (Table S5). Secondly, significant difference existed between two assemblies in terms of the HiC interaction heatmap. Dozens of gaps (72) were detected in the Nanopore assembly^8^, while less gaps (13) were found in the HiFi assembly (Fig. 1b). Thirdly, the HiFi assembly (99.57%) possessed higher chromosome anchoring rate compared with the Nanopore assembly (Table S5). Fourthly, the Nanopore assembly showed higher genome BUSCO score, while the HiFi assembly showed higher gene set BUSCO score (Table S5). Fifthly, the HiFi assembly showed higher mapping rates against all RNA-seq samples generated by two studies (Table S1). Finally, telomeres were undetectable in all chromosomes of the Nanopore assembly based on the monomer ‘TTAGG/CCTAA’, while telomeres were present in both ends of nine chromosomes and single end of the remaining one of the HiFi assembly (Table S5). Thus, the HiFi assembly showed advancement in contiguity compared with the Nanopore assembly, representing a near telomere-to-telomere (T2T) assembly of *M. alternatus*.

**Fig. 3:**
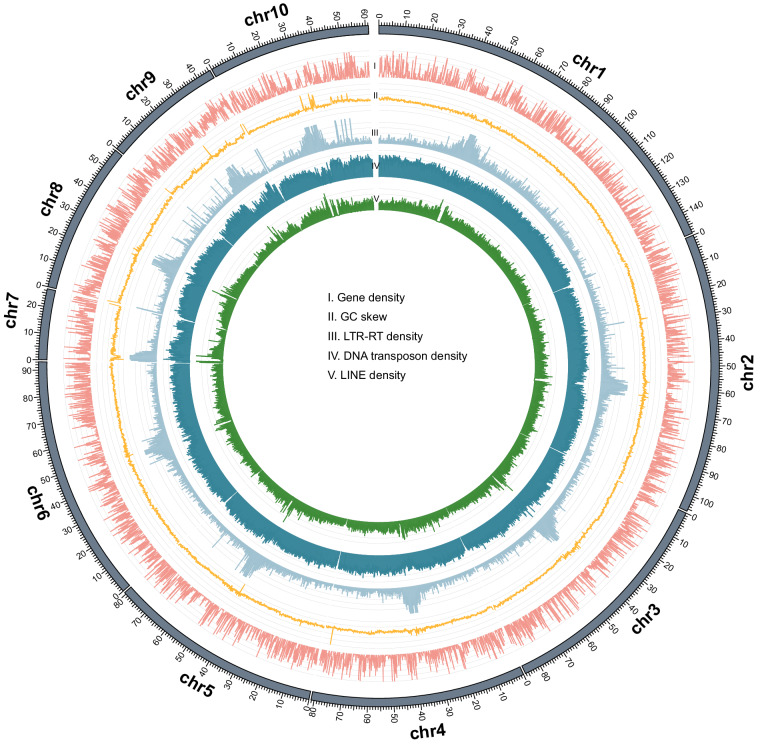
Overview of the genome landscape of the *Monochamus alternatus* genome assembly. In the Circos plot, the outmost track denotes the chromosomal ideograms (scale = 1 Mb). From the outer to the inner tracks, the density of protein-coding genes (I), GC skew (II), LTR-RTs (III), DNA transposons (IV), and LINEs (V) on each chromosome was calculated in nonoverlapping 100-kb windows and displayed.

The repeat sequences in *M. alternatus* were annotated using the RepeatMasker pipeline. In total, 58.23% of the *M. alternatus* genome was composed of repeat sequences. Among them, DNA transposons (35.81%) and long interspersed nuclear elements (LINEs, 9.45%) represent top two richest repeat types, and long terminal repeat retrotransposons (LTR-RTs), Penelope and short interspersed nuclear elements (SINEs) occupied 7.88%, 0.63% and 0.15% of genome sequence (Table 3). Based on the masked genome, we predicted protein-coding genes through combining three approaches, finally yielding 20,471 consensus protein coding genes (Table 2). BUSCO assessment showed that 97.6% of complete BUSCOs were present in the predicted gene set. Additionally, the canonical non-coding RNAs in *M. alternatus* were identified using different methods, including 1384 ribosomal RNAs (rRNAs), 540 transfer RNAs (tRNAs), 67 microRNAs (miRNAs), and 77 small nuclear RNAs (snRNAs) (Table 4). The landscape of *M. alternatus* genome assembly and gene annotations was presented as a Circos plot (Fig. 3). The functions of protein-coding genes were annotated against multiple database, such as SwissProt, InterPro, Pfam, Gene Ontology (GO), and Kyoto Encyclopedia of Genes and Genomes (KEGG). The results indicated that 98.04% of coding genes could be functionally annotated by at least one public database and transcriptome (Table 5), suggesting the high confidence of our gene annotation.

**Table 3:** Summary of repeat sequences in the genome assembly of *M. alternatus*.

Class	Number	Length	Percentage (%)
**LTR**			
Gypsy	29,600	24,111,649	3.05
Copia	1339	975,226	0.12
Other	98,671	37,100,109	4.71
**Non-LTR retroelements**			
LINE	213,558	74,577,309	9.45
SINE	12,366	1,166,742	0.15
Penelope	14,938	5,010,542	0.63
**DNA transposons**	896,596	282,736,732	35.81
**Simple repeats**	106,733	7,985,424	1.01
**Low complexity**	18,173	885,814	0.11
**Unclassified**	146,883	26,928,207	3.41
**Total**		459,696,228	58.23

**Table 4:** Summary of non-coding RNAs annotated in the *M. alternatus* genome.

Type	Copy number	Average length (bp)	Total length (bp)
miRNA	67	79.78	5345
tRNA	540	75.36	40,696
rRNA			
28S	39	2071.79	80,800
18S	53	1030.62	54,623
5.8S	101	119.16	3100
5S	1191	118	12,035
snRNA			
CD-box	20	143.35	2867
HACA-box	3	139	417
Splicing	54	148.52	8020

**Table 5:** Functional annotation of the predicted genes in *M. alternatus*.

Type	Number	Percentage (%)
SwissProt	10,527	51.42
RNA-seq	19,181	93.7
InterPro	16,317	79.71
Pfam	10,727	52.4
GO	8975	43.84
KEGG	12,426	60.7
Annotated	20,070	98.04
Unannotated	401	1.96
Total	20,471	100

**Fig. 4:**
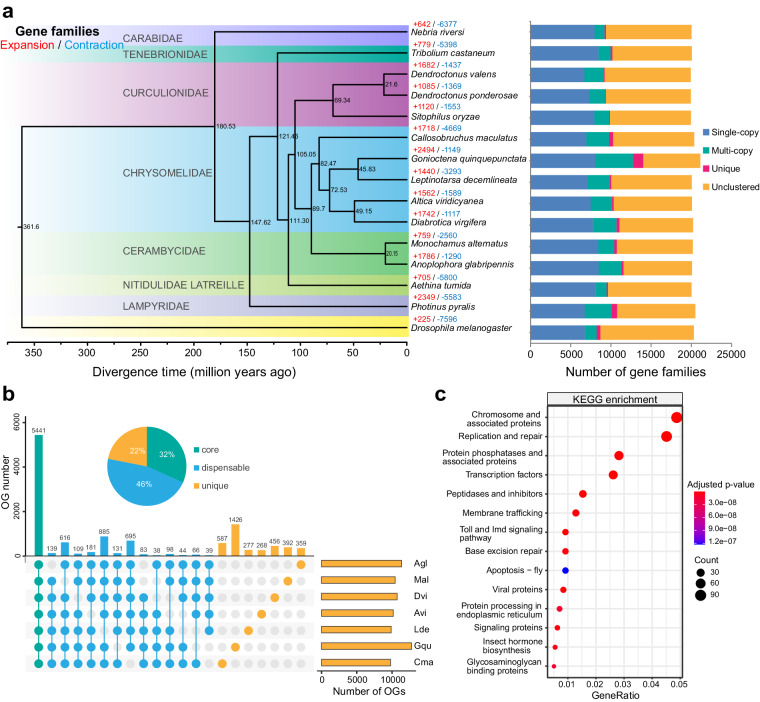
Phylogenetic tree of *Monochamus alternatus* and other Coleoptera insects and evolutionary analysis of gene families. **(a)** The phylogenetic tree of *M. alternatus* and other Coleoptera insects. The maximum likelihood tree was constructed using RAxML with *Drosophila melanogaster* as the outgroup based on 680 single-copy orthologs. The species divergence time was estimated using r8s. The numbers of expanded and contracted gene families revealed by CAFÉ analysis are shown above each species name. The stacked bar plots on the right represent the distribution of different types of genes in corresponding species, including single-copy, multi-copy, unique, and unclustered genes. **(b)** Upset plot showing the common and unique orthogroups identified in various Coleoptera insects. **(c)** KEGG enrichment analysis result of gene families that were significantly expanded in *M. alternatus* compared to the recent ancestor.

We selected thirteen coleopteran insect species to perform phylogenomic analysis. The phylogenetic tree was reconstructed from 680 strict single-copy orthologous genes using OrthoFinder. The results indicated that *M. alternatus* was most closely related to the Asian longhorned beetle *Anoplophora glabripennis*, and they diverged from each other approximately 25 million years ago (Mya) (Fig. 4a). CAFÉ analysis indicated that 759 gene families were significantly expanded in *M. alternatus* compared to the most recent common ancestor (Fig. 4a). We made a comparison of gene families across seven Coleoptera insects, and found that 5441 orthogroups (OGs) were highly conserved in these beetles, and 392 OGs were species-specific in *M. alternatus* (Fig. 4b). Functional enrichment analysis showed that the expanded gene families in *M. alternatus* were significantly enriched in many physiological processes, such as transcription factors (K09427; BH-adjusted p-value = 1.43 × 10^−12^), membrane trafficking (K21440; BH-adjusted p-value = 1.89 × 10^−9^), Toll and Imd signaling pathway (K20674; BH-adjusted p-value = 4.23 × 10^−5^), apoptosis (K20015; BH-adjusted p-value = 1.22 × 10^−7^), and insect hormone biosynthesis (K10719; BH-adjusted p-value = 3.99 × 10^−9^) (Fig. 4c).

## Methods

### Insect rearing, sample collection, and genome sequencing

Last instar larval *M. alternatus* were collected from host trees of *Pinus massoniana*, in Fuyang, Zhejiang province in late autumn in 2016. They were reared for approximately twenty generations in laboratory. The larva and pupa were fed on artificial diet in a 10-ml tube at a 12:12 h light:dark (L:D) cycle at 25 °C placed in a climate chamber. Fresh diet was provided every week^3^. The adults were fed on the fresh pine branches for sexual maturation and laid eggs under the back of pine logs (6–10 cm in diameter and 30 cm in length). One week later, the logs were debarked and the hatched larva were collected for next generation rearing. One newly emerged adult male without feeding was prepared for PacBio HiFi sequencing and Hi-C sequencing. The beetle’s gut was removed and body surface was cleaned by 75% ethanol to avoid microbial contamination.

Whole-Genome Sequencing (WGS) was used to estimate the genomic characteristics of *M. alternatus*. Illumina paired-end library was constructed and sequenced on Illumina HiSeq 2500 platform. A total of 43.05 Gb of clean data was generated (Table 1).

Genomic DNA for PacBio HiFi sequencing was prepared by the CTAB method and followed by purification with QIAGEN Blood & Cell Culture DNA Midi Kit (QIAGEN, USA) according to the manufactural procedure. The library was constructed Sequel II HiFi (CCS) method. SMRTbell target size libraries were constructed for sequencing according to the standard protocol of PacBio (Pacific Biosciences, CA USA) using 15 kb preparation solutions, and sequenced on PacBio Sequel II platform with Sequencing Primer V2 and Sequel II Binding Kit 2.0 in Grandomics Biosciences Co., Ltd (Wuhan, China).

To anchor hybrid scaffolds onto the chromosome, genomic DNA was extracted for the Hi-C library from the same beetle as HiFi. Then, we constructed the Hi-C library and obtained sequencing data via the Illumina DNBSEQ-T7 platform in Grandomics Biosciences Co., Ltd (Wuhan, China).

For transcriptome sequencing, the total RNA of each sample from development stage (egg, late larvae and early pupae) or adult tissue (gut, muscle, brain and trachea) was extracted using TRIzol (Thermo Fisher Scientific, USA). Paired-end libraries were constructed NEBNext®Ultra™ RNA Library Prep Kit for Illumina®(NEB, USA) following manufacturer’s recommendations. The library preparations were then paired-end sequenced on an Illumina HiSeq. 2500 platform. A total of 45.04 Gb of data was generated.

### Genome assembly and quality evaluation

To examine the genome feature of *M. alternatus*, GCE v1.0.2^17^ was used for k-mer frequency analysis with default parameters. To achieve high-quality genome assembly, Hifiasm (v0.16.1-r375)^18^ was used to *de novo* assemble the *M. alternatus* genome based on PacBio HiFi reads with default parameters. Then, the Hi-C reads were employed to scaffold contigs onto chromosomes through sorting, orientation, and ordering using Juicer v1.6^19^ and 3D-DNA v170123^20^ to generate the final version of genome assembly. Benchmarking Universal Single-Copy Orthologs (BUSCO v5.2.2)^16^ was performed to assess the quality of the genome assembly using the insecta_odb10 dataset.

### Identification of telomeres and centromeres

Both telomeres and centromeres were identified using quarTeT^21^. Telomeres were predicted using quarTeT TeloExplorer module with the parameters: ‘-c other -m 100’. Centromeres were predicted using quarTeT CentroMiner module with the parameters: ‘-n 100 -m 200 -s 0.8 -d 10 -e 0.00001 -g 50000 -i 100000 –trf 2 7 7 80 10 50 -r 3’. The chromosomal distribution of telomeres and centromeres was visualized using the R package chromoMap v4.1.12^22^.

### Chromosomal synteny analysis and identification of sex chromosomes

The whole-genome synteny analysis between *M. alternatus* and the closely related species *M. saltuarius* was performed using MUMmer v4.0.0beta2^23^ with default parameters. The chromosome that was syntenic with chrX in *M. saltuarius* was defined as the X chromosome in *M. alternatus*.

### Repeat sequence and non-coding RNA annotation

Firstly, a *de novo* repeat library was constructed by combining RepeatModeler v1.0.11 (http://www.repeatmasker.org/RepeatModeler) with LTR_retriever v2.9.0^24^, which integrates the LTR discovery result from LTR_finder v1.0.7^25^ and LTRharvest v1.5.9^26^. TRF v4.10.0^27^ was used for identification of tandem repeats. Then, the repeat sequences in the *M. alternatus* genome was identified and masked using RepeatMasker v4.0.7 (http://www.repeatmasker.org/RepeatMasker) against the species-specific *de novo* repeat library and RepBase library v26.03. Additionally, tRNAscan-SE^28^ was used to predict tRNA genes. Other non-coding RNAs such as rRNA, miRNA, and snRNA were annotated using INFERNAL v1.1^29^ through search against the Rfam database v9.1^30^.

### Gene prediction and functional annotation

To predict the protein-coding genes in the *M. alternatus* genome, we employed a strategy integrating *ab initio* prediction, homology searching and transcriptome-based approaches. For transcriptome-based prediction, HISAT v2.2.1^31^ was used to align the RNA-seq data to the reference genome, and StringTie v2.1.6^31^ was used for transcript assembly. Subsequently, TransDecoder v5.5.0 (https://github.com/TransDecoder/TransDecoder) was employed to estimate the potential open reading frames (ORFs). For the homology-based approaches, the protein sequences from seven Coleoptera insects and *Drosophila melanogaster* were downloaded from public database and GenomeThreader v1.7.1^32^ was used for homology search. For *ab initio* prediction, AUGUSTUS v3.4.0^33^ was employed for gene prediction based on the species-specific gene model. Finally, the EVidenceModeler pipeline^34^ was employed to generate a set of protein-coding genes through combining different sources of evidence. To maintain the confidence of predicted genes, we retained only gene models that had at least one supporting evidence from homologous proteins of closely related species, InterProScan domain and RNA-seq data. For functional annotation, we searched against the SwissProt protein database and InterPro database using DIAMOND v2.1.7^18^ (E-value = 1e-5) and InterProScan v5.21.60^35^, respectively. The assignment of KEGG orthology (KO) terms was conducted through search against the hidden Markov model (KOfam) database using KofamScan v1.3.0^36^ with default parameters.

### Reconstruction of phylogenetic tree of Coleoptera insect species

To perform phylogenomic analysis, the genome assemblies and gene annotations of thirteen Coleoptera insects and an outgroup *D. melanogaster* were retrieved from several public database, such as *A. glabripennis* (EnsemblMetazoa: Agla_1.0.48)^37^, *Aethina tumida* (NCBI RefSeq: GCF_024364675.1)^38^, *Altica viridicyanea* (NGDC GWH: GWHAMMQ00000000)^39^, *Callosobruchus maculatus* (NCBI GenBank: GCA_900659725.1)^40^, *Dendroctonus ponderosae* (NCBI GenBank: GCA_020466635.2)^41^, *Dendroctonus valens*^42^, *Diabrotica virgifera* (NCBI RefSeq: GCF_003013835.1)^43^, *Gonioctena quinquepunctata* (NCBI GenBank: GCA_018342105.1)^44^, *Leptinotarsa decemlineata* (i5k: OGSv1.2_GCF_000500325)^45^, *Nebria riversi*^46^, *Photinus pyralis* (NCBI RefSeq: GCF_008802855.1)^47^, *Sitophilus oryzae* (NCBI RefSeq: GCF_002938485.1)^48^, and *Tribolium castaneum* (EnsemblMetazoa: Tcas5.2.48)^49^. For each gene, only the longest transcript was kept for downstream analysis. OrthoFinder v2.5.4^50^ was performed to identify orthologs and paralogs using DIAMOND v2.1.7 with default parameters. To infer the phylogeny of these insects, multiple sequence alignments of single-copy orthologous genes were performed using MAFFT v7.490^51^ with default parameters. The alignment results were concatenated to form a super-sequence for each species and trimmed using trimAl v1.2^52^ with the “-gappyout” parameter. The optimal amino acid substitution model was estimated using ProtTest v3.4.2^53^. Then, RAxML v8.2.10^54^ was employed to construct the phylogenetic tree using the maximum likelihood method with LG + I + G + F model and 1000 bootstrap replicates. Species divergence age was adopted from the TimeTree^55^ database: *D. valens* vs *D. ponderosae* 21.6 Mya; *C. maculatus* vs *D. virgifera* 79–221 Mya; *T. castaneum* vs *D. melanogaster* 195–361.6 Mya; and *A. glabripennis* vs *L. decemlineata* 89.7–220.9 Mya. R8s v1.81^56^ was used for calibrating the species divergence time. The species tree was visualized using FigTree v1.4.2.

### Gene family expansion and contraction

The count matrix of gene family for each species was obtained from OrthoFinder analysis. The matrix table and the ultrametric tree were taken as inputs to analyze the expansion and contraction of gene families using CAFE v4.2.1^57^.

## Data Records

Raw data from PacBio HiFi (CRR1002983)^58^, Hi-C (CRR1002984)^59^ and Illumina (CRR1002982)^60^ genome sequencing and RNA-seq data (CRR1003137-CRR1003143)^61–67^ have been deposited in the Genome Sequence Archive (GSA, https://ngdc.cncb.ac.cn/gsa) at the National Genomic Data Center (NGDC)^68^. The genome assembly has been deposited in the Genome Warehouse (GWH, https://ngdc.cndb.ac.cn/gwh)^69^ at NGDC under the accession number of GWHEQWP00000000. All data were associated with the BioProject PRJCA022378. The genome sequence and raw reads have also been deposited in GenBank (JBBBDW000000000.1^70^) and Sequence Read Archive (SRA, SRR28248702^71^ for PacBio HiFi, SRR28248701^72^ for Hi-C, SRR28248703^73^ for Illumina, and SRR28248694-SRR28248700^74–80^ for RNA-seq data) at National Center for Biotechnology Information (NCBI) under BioProject PRJNA1084890.

## Technical Validation

Two distinct methods were employed to assess the completeness and accuracy of the *M. alternatus* genome. First, employing the insecta_odb10 datasets, the BUSCO analysis demonstrated successful identification of 99.0% of core genes as complete. Second, the realignment of Illumina genome sequencing and RNA-seq reads to the *M. alternatus* genome resulted in mapping rates of 98.32% and 95.53%, respectively. To further appraise the comprehensiveness and accuracy of gene prediction, BUSCO analysis was conducted based on the Insecta datasets, yielding a complete BUSCO score of 97.6%.

### Supplementary information

Supplementary information

## Data Availability

No specific script was utilized in this study. The codes and pipelines used for genome sequencing data analysis were performed following the instructions of corresponding bioinformatics tools. The version and parameters of the software have been included in the Methods section.
