# Supplementary material for: A near-complete genome assembly of Monochamus alternatus a major vector beetle of pinewood nematode
Source: Sci Data. 2024 Mar 26;11:312. doi: 10.1038/s41597-024-03150-1 (PMC10966038; doi:10.1038/s41597-024-03150-1)
Supplement: Supplementary file 1 — Supplementary information [file 41597_2024_3150_MOESM1_ESM.docx]

**Supplementary information**

**A near-complete genome assembly of *Monochamus alternatus* a major vector beetle of pinewood nematode**

Longsheng Xing^1, #^, Bo Liu^2, #^, Dunyang Yu^1, #^, Xuan Tang^1^, Jianghua Sun^1, *^, Bin Zhang^1, *^

^1^ College of Life Science/Hebei Basic Science Center for Biotic Interactions, Institute of Life Science and Green Development, Hebei University, Baoding, 071002, China

^2^ Shenzhen Branch, Guangdong Laboratory for Lingnan Modern Agriculture, Genome Analysis Laboratory of the Ministry of Agriculture and Rural Affairs, Agricultural Genomics Institute at Shenzhen, Chinese Academy of Agricultural Sciences, Shenzhen 518120, China

**Supplementary Information Index**

Figure S1 2

Table S1 3

Table S2 3

Table S3 8

Table S4 9

Table S5 9

**Supplementary figure**


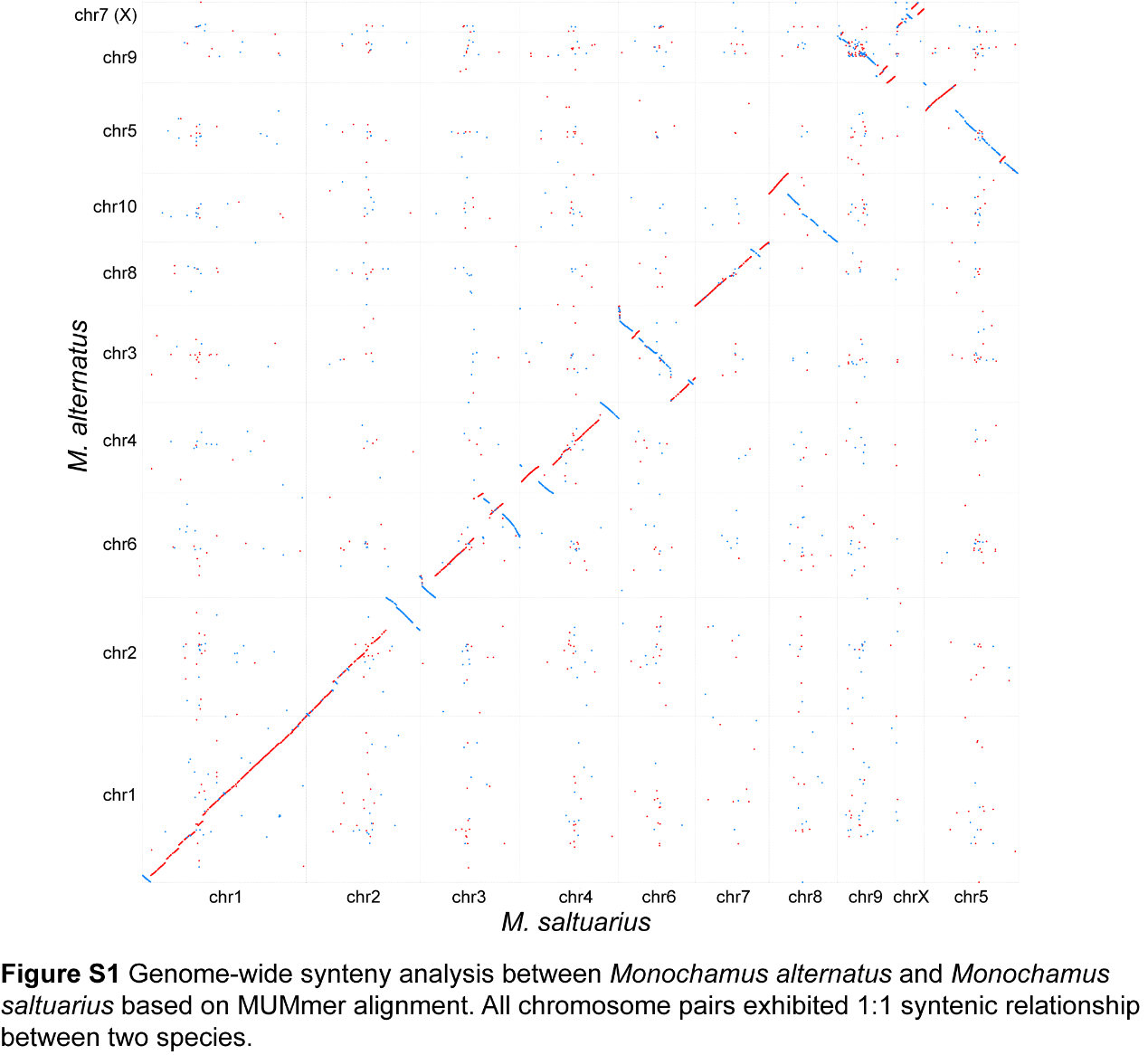


**Figure S1**: Genome-wide synteny analysis between *Monochamus alternatus* and *Monochamus saltuarius* based on MUMmer alignment. All chromosome pairs exhibited 1:1 syntenic relationship between two species.

| **Supplementary tables**  **Table S1:** Mapping rates of Illumina genome sequencing and RNA-seq data in *Monochamus alternatus* | | | | |
| --- | --- | --- | --- | --- |
| **Sample** | **Sequence type** | **Mapping rate** | | **Source** |
|  |  | **Nanopore assembly** | **HiFi assembly** |  |
| DSW42578-S_L3 | WGS | - | 98.32% | This study |
| SRR26116071 | RNA-seq | 89.68% | 93.11% | (Gao et al., 2024) |
| SRR26116072 | RNA-seq | 89.15% | 93.20% | (Gao et al., 2024) |
| SRR26116073 | RNA-seq | 90.68% | 93.74% | (Gao et al., 2024) |
| Mal_Tr | RNA-seq | 72.16% | 95.09% | This study |
| Mal_brain | RNA-seq | 88.27% | 94.24% | This study |
| Mal_early_pupa | RNA-seq | 92.63% | 95.25% | This study |
| Mal_egg_soft | RNA-seq | 94.83% | 97.05% | This study |
| Mal_gut | RNA-seq | 92.27% | 95.06% | This study |
| Mal_late_larvae | RNA-seq | 93.23% | 96.07% | This study |
| Mal_muscle | RNA-seq | 55.70% | 95.98% | This study |

| **Table S2:** Summary of genome size and contig N50 of publicly available chromosome-scale genome assemblies of Coleoptera insects | | | |
| --- | --- | --- | --- |
| **Accession** | **Contig N50 size (Mb)** | **Genome size (Mb)** | **Species** |
| this study | 55.988924 | 789.497424 | *Monochamus alternatus* |
| GCA_932526585.2 | 29.486216 | 1030.568945 | *Philonthus cognatus* (beetles) |
| GCA_951802165.1 | 26.378788 | 489.453138 | *Adalia decempunctata* (beetles) |
| GCA_029229535.1 | 24.536916 | 449.895081 | *Diorhabda carinata* (beetles) |
| GCA_921294245.1 | 24.416959 | 585.278335 | *Brassicogethes aeneus* (rapeseed pollen beetle) |
| GCF_026250575.1 | 23.459757 | 415.385902 | *Diorhabda carinulata* (beetles) |
| GCF_914767665.1 | 22.915403 | 425.524972 | *Harmonia axyridis* (beetles) |
| GCA_905340355.1 | 21.086067 | 424.679337 | *Rhagonycha fulva* (beetles) |
| GCA_935413205.1 | 19.113408 | 713.374358 | *Polydrusus cervinus* (beetles) |
| GCA_910592335.1 | 18.317171 | 475.288177 | *Adalia bipunctata* (two-spotted ladybird beetle) |
| GCA_911387805.1 | 17.823022 | 445.936792 | *Cantharis rustica* (beetles) |
| GCF_907165205.1 | 16.512651 | 398.849173 | *Coccinella septempunctata* (seven-spotted ladybird) |
| GCA_030157265.1 | 16.486265 | 170.052028 | *Latheticus oryzae* (longheaded flour beetle) |
| GCA_018691245.2 | 16.438241 | 231.406857 | *Ips nitidus* (beetles) |
| GCA_031307605.1 | 16.193484 | 241.845558 | *Tribolium castaneum* (red flour beetle) |
| GCA_911728475.2 | 15.821749 | 705.1468 | *Pterostichus madidus* (beetles) |
| GCA_911728435.2 | 15.031106 | 428.077388 | *Apoderus coryli* (hazel leaf roller) |
| GCA_937662695.2 | 14.028202 | 919.111547 | *Halyzia sedecimguttata* (orange ladybird) |
| GCA_963082815.1 | 13.836834 | 803.481935 | *Melanotus villosus* (beetles) |
| GCA_023509865.1 | 12.989 | 636.265192 | *Trypoxylus dichotomus* (beetles) |
| GCA_905333025.2 | 12.970676 | 249.414446 | *Pyrochroa serraticornis* (beetles) |
| GCA_947534325.1 | 12.663309 | 695.910864 | *Agonum fuliginosum* (beetles) |
| GCF_026230105.1 | 12.487216 | 456.219552 | *Diorhabda sublineata* (beetles) |
| GCA_026230145.1 | 11.993826 | 485.337276 | *Diorhabda elongata* (beetles) |
| GCA_030157275.1 | 11.790235 | 236.991183 | *Cynaeus angustus* (beetles) |
| GCF_024364675.1 | 11.74249 | 259.984281 | *Aethina tumida* (small hive beetle) |
| GCA_963583935.1 | 11.653919 | 542.638604 | *Diaperis boleti* (beetles) |
| GCA_958510845.1 | 10.907 | 397.814522 | *Cantharis nigra* (beetles) |
| GCA_963170105.1 | 10.697386 | 332.197494 | *Cantharis lateralis* (beetles) |
| GCA_949152465.1 | 10.50509 | 348.304867 | *Cantharis flavilabris* (beetles) |
| GCA_947369205.1 | 10.477904 | 355.331611 | *Cantharis rufa* (beetles) |
| GCA_932274525.1 | 9.879682 | 777.254275 | *Podabrus alpinus* (beetles) |
| GCA_958510865.1 | 9.66 | 769.912521 | *Myrrha octodecimguttata* (beetles) |
| GCA_946251905.1 | 9.642473 | 500.560161 | *Cryptocephalus moraei* (beetles) |
| GCA_949126875.1 | 9.517382 | 534.692516 | *Lochmaea capreae* (willow leaf beetle) |
| GCA_030068095.1 | 9.069096 | 738.372295 | *Anthonomus grandis* *thurberiae* (boll weevil) |
| GCA_949768715.1 | 8.885058 | 282.75556 | *Dascillus cervinus* (orchid beetle) |
| GCA_951394225.1 | 8.784621 | 890.55349 | *Harpalus rufipes* (beetles) |
| GCA_029298725.1 | 8.578702 | 342.674785 | *Gnatocerus cornutus* (beetles) |
| GCA_939628115.1 | 8.487211 | 269.169934 | *Tribolium freemani* (beetles) |
| GCA_943142095.1 | 8.331656 | 911.917119 | *Ophonus ardosiacus* (beetles) |
| GCA_963082785.1 | 8.244937 | 671.08243 | *Philonthus spinipes* (beetles) |
| GCA_949128085.1 | 8.085049 | 479.646749 | *Cetonia aurata* (beetles) |
| GCF_917563875.1 | 7.930419 | 2533.404242 | *Diabrotica virgifera virgifera* (western corn rootworm) |
| GCA_947359425.1 | 7.715647 | 336.783501 | *Lagria hirta* (beetles) |
| GCA_951805005.1 | 7.68556 | 870.474623 | *Othius punctulatus* (beetles) |
| GCA_963082805.1 | 7.643588 | 377.570513 | *Hemicrepidius niger* (beetles) |
| GCA_933228885.1 | 7.480091 | 235.131629 | *Leistus spinibarbis* (beetles) |
| GCA_958510855.1 | 7.455203 | 479.857303 | *Rhagonycha lutea* (beetles) |
| GCA_024712935.1 | 7.451212 | 1008.323064 | *Leptinotarsa decemlineata* (Colorado potato beetle) |
| GCA_959613385.1 | 7.348763 | 580.588114 | *Geotrupes spiniger* (beetles) |
| GCA_917563865.1 | 7.248987 | 135.120912 | *Phyllotreta cruciferae* (beetles) |
| GCA_949316355.1 | 7.036267 | 375.583928 | *Bruchidius siliquastri* (beetles) |
| GCF_022605725.1 | 6.865999 | 697.452952 | *Anthonomus grandis grandis* (boll weevil) |
| GCA_949128115.1 | 6.778 | 757.84431 | *Anaspis maculata* (beetles) |
| GCA_022388445.1 | 6.737797 | 478.14507 | *Zophobas atratus* (beetles) |
| GCA_944452925.2 | 6.296 | 1423.453393 | *Chrysolina oricalcia* (beetles) |
| GCA_963082715.1 | 5.994626 | 1253.243932 | *Harpalus rubripes* (beetles) |
| GCA_935421215.2 | 5.92969 | 1656.884372 | *Melolontha melolontha* (cockchafer) |
| GCA_963243735.1 | 5.881602 | 671.316925 | *Neocrepidodera transversa* (beetles) |
| GCA_910589415.1 | 5.740145 | 544.256285 | *Malachius bipustulatus* (malachite beetle) |
| GCA_028583605.1 | 5.669615 | 538.90235 | *Dastarcus helophoroides* (beetles) |
| GCA_958507055.1 | 5.465325 | 639.299739 | *Crioceris asparagi* (common asparagus beetle) |
| GCA_947389935.1 | 5.283 | 292.354128 | *Agrilus cyanescens* (beetles) |
| GCA_025584915.1 | 4.817785 | 682.219095 | *Monochamus saltuarius* (beetles) |
| GCA_963584125.1 | 4.745322 | 236.737571 | *Plagiodera versicolora* (willow leaf beetle) |
| GCA_910593695.2 | 4.567902 | 1083.870417 | *Ocypus olens* (beetles) |
| GCA_950111635.1 | 4.545743 | 692.286867 | *Agelastica alni* (beetles) |
| GCA_929113105.1 | 4.463 | 1578.523859 | *Agrypnus murinus* (beetles) |
| GCA_949320105.2 | 4.358255 | 508.975589 | *Crepidodera aurea* (beetles) |
| GCA_029378335.1 | 4.341085 | 291.300295 | *Agrilus mali* (beetles) |
| GCA_936432065.2 | 4.27209 | 2021.580433 | *Rutpela maculata* (beetles) |
| GCA_963422195.1 | 4.259 | 253.974633 | *Carabus problematicus* (beetles) |
| GCA_019155225.1 | 4.094517 | 305.13347 | *Tribolium confusum* (confused flour beetle) |
| GCA_958502065.1 | 4.093593 | 980.590408 | *Chrysolina americana* (beetles) |
| GCA_963583905.1 | 3.913043 | 1616.911602 | *Stenurella melanura* (beetles) |
| GCA_963170755.1 | 3.724788 | 1584.161395 | *Amphimallon solstitiale* (beetles) |
| GCA_951805265.1 | 3.604651 | 181.134879 | *Schizotus pectinicornis* (beetles) |
| GCA_033060865.1 | 3.575657 | 520.705759 | *Dorcus hopei* (beetles) |
| GCA_917834065.1 | 3.53848 | 674.95087 | *Ceutorhynchus assimilis* (cabbage seed weevil) |
| GCA_963576565.1 | 3.520329 | 1286.186002 | *Aspidapion aeneum* (beetles) |
| GCA_950108345.1 | 3.462109 | 236.723918 | *Malthinus flaveolus* (beetles) |
| GCA_947310635.1 | 3.374168 | 492.378077 | *Leptodirus hochenwartii* (beetles) |
| GCA_944039245.1 | 3.131785 | 256.740106 | *Nebria salina* (beetles) |
| GCA_963457615.1 | 3.077846 | 202.291714 | *Nicrophorus investigator* (beetles) |
| GCA_947563755.1 | 2.848502 | 891.301393 | *Lochmaea crataegi* (beetles) |
| GCA_947425015.1 | 2.834613 | 674.098515 | *Pterostichus niger* (beetles) |
| GCA_963576655.1 | 2.815411 | 369.770545 | *Gastrophysa polygoni* (beetles) |
| GCA_958336395.1 | 2.792496 | 647.686976 | *Ctenicera cuprea* (beetles) |
| GCA_918026865.1 | 2.676005 | 132.261818 | *Phyllotreta striolata* (beetles) |
| GCA_958510875.1 | 2.494558 | 906.444856 | *Endomychus coccineus* (beetles) |
| GCA_944738965.1 | 2.458479 | 241.934194 | *Nebria brevicollis* (beetles) |
| GCA_949748235.1 | 2.446278 | 147.483955 | *Platypus cylindrus* (beetles) |
| GCA_958298965.1 | 2.238999 | 718.322849 | *Chrysolina haemoptera* (beetles) |
| GCA_944588485.1 | 2.238738 | 1199.998149 | *Phosphuga atrata* (black snail beetle) |
| GCA_949788335.1 | 2.186765 | 127.540636 | *Salpingus planirostris* (beetles) |
| GCA_918026855.4 | 1.95033 | 869.734947 | *Phaedon cochleariae* (mustard beetle) |
| GCA_963576515.1 | 1.867949 | 371.011342 | *Cryptocephalus primarius* (beetles) |
| GCA_963576705.1 | 1.537882 | 1392.699176 | *Polydrusus tereticollis* (beetles) |
| GCA_918026665.1 | 1.45954 | 1607.257046 | *Diabrotica balteata* (beetles) |
| GCA_958336345.1 | 1.432485 | 470.89872 | *Dorcus parallelipipedus* (lesser stag beetle) |
| GCA_951812935.1 | 1.363883 | 479.410748 | *Hermaeophaga mercurialis* (beetles) |
| GCA_927349885.1 | 1.287164 | 1181.805965 | *Psylliodes chrysocephala* (beetles) |
| GCA_958502075.1 | 1.248716 | 624.046445 | *Orchestes rusci* (beetles) |
| GCA_951812265.1 | 1.227 | 575.240612 | *Taphrorychus bicolor* (beetles) |
| GCA_022388455.1 | 1.044995 | 300.426119 | *Tribolium freemani* (beetles) |
| GCA_947652605.1 | 0.911544 | 516.518598 | *Elmis aenea* (beetles) |
| GCA_011033045.2 | 0.889999 | 415.312956 | *Harmonia axyridis* (beetles) |
| GCA_013421045.1 | 0.813984 | 851.227647 | *Propylea japonica* (beetles) |
| GCA_963556235.1 | 0.622935 | 177.342891 | *Cryptophagus acutangulus* (beetles) |
| GCA_023690525.1 | 0.369487 | 1415.113966 | *Holotrichia oblita* (beetles) |
| GCA_019049505.1 | 0.299666 | 2051.389195 | *Pachyrhynchus sulphureomaculatus* (beetles) |
| GCA_958510825.1 | 0.212382 | 648.296476 | *Brachypterus glaber* (beetles) |
| GCA_940337035.1 | 0.201347 | 3159.863714 | *Agriotes lineatus* (beetles) |
| GCF_000002335.3 | 0.073049 | 165.944485 | *Tribolium castaneum* (red flour beetle) |
| GCA_027562985.1 | 0.035526 | 353.918979 | *Chrysomela aeneicollis* (beetles) |
| GCA_002278615.1 | 0.005623 | 280.876046 | *Pogonus chalceus* (beetles) |

| **Table S3:** Identification result of telomeres in the *M. alternatus* genome assembly | | | | | | |
| --- | --- | --- | --- | --- | --- | --- |
| **Chr** | **Chromosome length** | **Status** | **Left repeat times** | **Left direction** | **Right repeat times** | **Right direction** |
| chr1 | 148415838 | both | 582 | + | 2946 | - |
| chr2 | 105897448 | both | 1228 | + | 1209 | - |
| chr3 | 86209243 | both | 1143 | + | 311 | - |
| chr4 | 81173492 | both | 1192 | + | 329 | - |
| chr5 | 80724879 | both | 318 | + | 889 | - |
| chr6 | 93112755 | both | 324 | + | 611 | - |
| chr7 (X) | 26691667 | left | 2258 | + | 0 |  |
| chr8 | 57178267 | both | 287 | + | 960 | - |
| chr9 | 45529815 | both | 444 | + | 392 | - |
| chr10 | 61192587 | both | 466 | + | 677 | - |

| **Table S4:** Summary of centromeric regions identified in the *M. alternatus* genome assembly | | | | | | |
| --- | --- | --- | --- | --- | --- | --- |
| **Chr** | **Start** | **End** | **Length** | **TRlength** | **TRcoverage** | **Region score** |
| chr1 | 46862870 | 50708016 | 3845147 | 438642 | 11.41% | 0.114076813 |
| chr2 | 26246973 | 28983672 | 2736700 | 257387 | 9.41% | 0.094050168 |
| chr3 | 69169245 | 69398494 | 229250 | 15723 | 6.86% | 0.068584814 |
| chr4 | 34858554 | 36388886 | 1530333 | 134699 | 8.80% | 0.088019462 |
| chr5 | 46886216 | 47672177 | 785962 | 73540 | 9.36% | 0.093566984 |
| chr6 | 44422681 | 47630428 | 3207748 | 262252 | 8.18% | 0.081755824 |
| chr7 (X) | 18706357 | 18828742 | 122386 | 11129 | 9.09% | 0.090934347 |
| chr8 | 27156539 | 27469656 | 313118 | 60837 | 19.43% | 0.194294784 |
| chr9 | 36454251 | 36631436 | 177186 | 35594 | 20.09% | 0.20088608 |
| chr  10 | 45738177 | 45970295 | 232119 | 142245 | 61.28% | 0.61281331 |

| **Table S5:** Summary of genome metrics for two versions of genome assemblies | | |
| --- | --- | --- |
| **Metrics** | **Published version (Nanopore)** | **This study version (PacBio HiFi)** |
| Contig N50 (Mb) | 15.77 | 55.99 |
| Scaffold N50 (Mb) | 82.97 | 86.21 |
| Number of gaps | 72 | 13 |
| Anchoring rate (%) | 98.21 | 99.57 |
| Complete BUSCOs of genome assembly | 99.70% | 99.00% |
| Complete BUSCOs of gene annotation | 96.70% | 97.60% |
| Presence of telomeres | Absent in all chromosomes | Both ends present in nine chromosomes, and single end present in one chromosome |
| Presence of centromeres | Present in all chromosomes | Present in all chromosomes |
